# Editorial: Women in psychiatry 2025: addictive disorders

**DOI:** 10.3389/fpsyt.2026.1944447

**Published:** 2026-08-05

**Authors:** Stefania Chiapini, Annagiulia Di Trana

**Affiliations:** 1 UniCamillus International University of Medical Sciences, Rome, Italy; 2 National Centre on Addiction and Doping, National Institute of Health, Rome, Italy

**Keywords:** addictive daydreaming scale, addictive disorders, craving measurement, mental wellbeing, psychopharmacology (PSP), women psychiatrists

Addictive disorders remain among the leading contributors to the global burden of disease, with profound consequences for mental wellbeing, physical health, and society. Increasing recognition of the complex interactions between biological vulnerability, psychological processes, social determinants, and gender has transformed addiction research from a disorder-specific perspective into a multidisciplinary field. At the same time, the scientific community has become increasingly aware that diversity among researchers enriches innovation and broadens the range of questions addressed. Against this background, the *Women in Psychiatry 2025: Addictive Disorders* R*esearch* T*opic* was conceived to highlight the scientific contributions of women researchers while advancing contemporary knowledge across the spectrum of addictive disorders. The Research Topic welcomes research spanning substance use disorders, behavioural addictions, dual diagnosis, prevention, assessment, and treatment, reflecting the breadth and interdisciplinarity required to address addiction in modern psychiatry.

The four contributions assembled in this Research Topic illustrate the diversity of contemporary addiction research while converging on a common objective: improving the understanding of factors that influence the onset, maintenance, assessment, and management of addictive behaviours. Rather than focusing on a single disorder or intervention, the studies collectively explore psychological resilience, innovative assessment methods, emerging behavioural addictions, and clinically relevant therapeutic challenges. Together, they underscore the importance of moving beyond traditional diagnostic boundaries towards more personalized, evidence-based, and patient-centred approaches.

One major theme emerging from the Research Topic concerns the psychological mechanisms that underlie vulnerability and recovery. The study from Gliksberg et al. highlights the protective role of meaning in life as a central psychological construct capable of interrupting the reciprocal relationship between psychological distress and addictive behaviours. By proposing an integrative framework linking existential and mental wellbeing, and addiction, the authors extend current theoretical models and encourage clinicians to consider positive psychological resources alongside symptom reduction. This perspective aligns with growing interest in recovery-oriented psychiatry, where strengthening resilience and personal meaning complements conventional therapeutic approaches.

A second contribution addresses methodological innovation in addiction assessment. The brief research report *Do response times add to self-reported craving? A secondary analysis of a neuromodulation trial* investigates whether response latency can provide additional information beyond traditional self-reported craving measures. Although craving remains a central construct in addiction research, its subjective nature presents important methodological limitations. Exploring behavioural indicators that complement self-report may improve the sensitivity of outcome measures and enhance the evaluation of novel interventions, including neuromodulation. Such work reflects the broader movement towards digital phenotyping and multimodal assessment strategies capable of capturing the complexity of addictive processes.

The Research Topic also contributes to the expanding field of behavioural addictions through the original research article *Introducing the Addictive Daydreaming Scale: development and Polish validation of the ADS-20 and ADS-5*. As excessive or maladaptive daydreaming receives increasing clinical attention, reliable psychometric instruments become essential for advancing epidemiological, clinical, and neurobiological research. The development and validation of standardized assessment tools represent a critical first step in characterizing emerging behavioural addictions, differentiating pathological from normative experiences, and facilitating international comparisons across populations. This study therefore provides an important methodological foundation for future investigations into an increasingly recognized but still understudied phenomenon.

Completing the Research Topic, the case report on trazodone as a possible treatment for psychiatric adverse events related to anaplastic lymphoma kinase inhibitors highlights the close relationship between addiction psychiatry, psychopharmacology, and consultation-liaison psychiatry. Although not centred on a substance use disorder itself, the report demonstrates the complexity of managing psychiatric symptoms arising during treatment for severe medical illnesses and illustrates how careful psychopharmacological decision-making can improve patient outcomes. The inclusion of clinically focused reports such as this broadens the scope of addiction psychiatry by emphasizing that psychiatric expertise extends beyond diagnostic categories to encompass individualized management of complex patients with multiple medical and psychological needs.

Taken together, these contributions emphasize several broader trends shaping contemporary addiction research. Advances in measurement science are providing increasingly sophisticated tools to assess constructs that have traditionally relied on subjective reporting. This field continues to expand beyond substance-related disorders to include behavioural addictions whose mechanisms may share common neuropsychological pathways. Finally, personalized approaches to assessment and treatment are becoming central to psychiatric practice, acknowledging the heterogeneity of patients’ experiences and therapeutic needs.

An equally important aspect of this Research Topic is its commitment to promoting the visibility and leadership of women in psychiatric research. Despite substantial progress, women remain underrepresented in many areas of scientific leadership and academic medicine. Initiatives such as the Women in Psychiatry series provide valuable opportunities to showcase scientific excellence while fostering greater diversity within the research community. Increasing representation is not merely a matter of equity; diverse research teams generate broader scientific perspectives, ask novel questions, and ultimately strengthen the evidence base informing clinical care.

As addiction research continues to evolve, future investigations should further explore longitudinal trajectories, culturally sensitive interventions, digital technologies, precision psychiatry, and implementation science. Greater integration of biological markers with psychological and social determinants may facilitate more individualized prevention and treatment strategies. Equally important will be maintaining a commitment to inclusivity—both in the populations studied and among the researchers generating new knowledge.

